# ‘A No‐Brainer, but Not a Simple Decision’: A Qualitative Study of Patient Experiences of Tissue Donation for Research in Rare Craniospinal Tumour Pathways

**DOI:** 10.1111/hex.70839

**Published:** 2026-08-22

**Authors:** Gerard Mawhinney, Simona Fourie, Simon Leedham, Helen Higham, Olaf Ansorge

**Affiliations:** ^1^ Nuffield Department of Clinical Neurosciences University of Oxford Oxford England UK; ^2^ Radcliffe Department of Medicine University of Oxford Oxford England UK; ^3^ Nuffield Department of Medicine University of Oxford Oxford England UK

**Keywords:** craniospinal tumours, digital consent support, informed consent, patient experience, qualitative research, rare cancer, tissue donation

## Abstract

**Background:**

Tissue donation is central to rare tumour research, yet consent is often sought when patients are managing diagnosis, major surgery and uncertainty. Little is known about how people with rare craniospinal tumours experience tissue‐donation consent or what support they consider useful.

**Objective:**

To explore patient experiences of tissue‐donation consent in rare craniospinal tumour pathways and identify implications for patient‐centred, staged and relational consent support.

**Design:**

Qualitative interview study following a national online survey.

**Setting and Participants:**

Eight adults from a self‐selected volunteer subsample of respondents to a preceding national survey completed online semi‐structured interviews using a survey‐informed interview guide. All 50 survey respondents were invited to volunteer for interview; 13 expressed interest, 8 ultimately provided informed consent, and all 8 were interviewed. No further participant selection was undertaken by the research team.

**Results:**

Four themes were generated beneath the overarching analytic thread of making sense of tissue donation across the rare tumour pathway: tissue donation as something good coming from something bad; tissue donation during shock and overload; making sense of tissue donation over time; and trust and support around tissue donation. Participants described the need for trusted information support that extended beyond leaflets while complementing clinician‐led discussion.

**Conclusions:**

Within this small, self‐selected volunteer sample, participants' accounts suggest that tissue‐donation consent may be better supported as a staged, relational and revisitable process rather than treated as a single procedural event.

**Lived Experience or Public Contribution:**

People with lived experience informed survey development and testing. Interview participants contributed detailed accounts that shaped the consent‐support implications presented in this manuscript.

## 1 Introduction

Primary craniospinal tumours are rare, heterogeneous and frequently managed through complex specialist pathways. In this study, ‘craniospinal tumour pathways’ is used pragmatically to describe specialist neurosurgical and spinal/axial sarcoma pathways for rare primary tumours involving the skull base, spine or related axial skeleton, rather than the radiotherapy use of ‘craniospinal’ to describe treatment fields. Translational progress in these conditions depends on access to high‐quality tumour tissue and linked clinical data. Tissue donation is therefore central to research feasibility, enabling molecular characterisation, biomarker discovery, genomic analysis and future therapeutic development [1].

Yet tissue donation is usually discussed at a time when patients are also managing diagnosis, major surgery, prognostic uncertainty, and treatment planning. These circumstances raise ethical, relational and practical questions about how research participation is introduced, how information is understood, how voluntariness is preserved and how patients and families can be supported [2, 3, 4].

Existing evidence suggests that many patients are willing to donate tissue for research, especially where tissue is surplus to clinical requirements [2, 3]. Oncology biobanking studies also show that willingness may not translate into an actual opportunity to participate: clinician‐initiated opt‐in processes can lose willing participants through inconsistent offering, incomplete registration and poor recall of consent [5, 6]. Evidence from cancer clinical trials similarly shows that satisfaction with consent and self‐perceived informedness can coexist with substantial gaps in understanding [7]. The coexistence of high willingness and imperfect understanding is therefore not, in itself, a novel observation; the relevant question for this study is how that tension is experienced and supported within rare craniospinal tumour pathways.

Related qualitative literature in brain tumour donation also demonstrates that tissue donation can carry meanings of altruism, purpose, control and legacy. Adults with high‐grade brain cancer consenting to post‐mortem brain donation have described donation as meaningful and empowering [8], while parents who donated a child's high‐grade glioma tissue post‐mortem emphasised altruism alongside the need for clear information about donation procedures [9]. Rare‐cancer research literature similarly emphasises meaningful patient engagement, trust, respect, inclusivity and sustained relationships with research teams [10, 11]. However, these studies concern different donation contexts or broader research engagement. Evidence remains limited on living adults navigating tissue‐donation consent within rare craniospinal surgical pathways and on the pathway features that may support a good consent experience.

A preceding national survey within this programme found generally positive experiences but identified practical opportunities to strengthen consent support through appropriate timing, clear and revisitable information, opportunities for questions and resources that support family‐inclusive decision‐making [1]. The present qualitative study was designed to explore these experiences in greater depth. Understanding tissue donation from the perspective of patient experience is necessary if consent pathways are to be ethically robust, practically feasible and responsive to the circumstances in which decisions are made.

This study aimed to explore how people with rare craniospinal tumours experience tissue‐donation consent within specialist care pathways, and to identify implications for patient‐centred, staged and relational consent support.

## 2 Methods

### 2.1 Theoretical Position and Study Design

This qualitative interview study formed the qualitative follow‐up phase of a wider sequential mixed‐methods programme, following a national online survey of people with lived experience of primary craniospinal tumours [1, 12]. This study was not designed to test a formal theory; rather, it used an inductive qualitative approach to generate a practice‐relevant, patient‐experience account of tissue‐donation consent in rare craniospinal tumour pathways [13]. Semi‐structured interviews enabled focused exploration while allowing participants to describe experiences in their own terms. The survey‐informed interview schedule used open‐ended prompts to elicit rich experiential accounts [14]. Data were analysed using reflexive thematic analysis to identify patterns of shared meaning across participants' accounts. Reporting was reviewed against COREQ, with the completed checklist provided as Supplementary File 2 [15].

### 2.2 Setting, Sampling and Recruitment of Participants

Participants were recruited from respondents to the preceding national online survey. The survey was promoted across UK brain tumour and sarcoma charity networks, social media outlets and patient‐facing digital platforms. Eligible survey participants were adults aged 18 years or older, living in the United Kingdom, with lived experience of a primary tumour of the brain or spine, able to provide informed consent, communicate in English and access the internet‐enabled survey.

At the end of the survey, all respondents were invited to volunteer for a short online interview by selecting an opt‐in box and providing an email address. The invitation made clear that interview participation was optional and required separate consent.

Fifty participants completed the online survey. Thirteen respondents expressed interest in being contacted about an interview, eight subsequently provided informed consent, and all eight were interviewed. Reasons why five individuals who initially expressed interest did not proceed were not collected. No further participant selection was undertaken by the research team according to diagnosis, donation status, participant characteristics or expressed views. The interview group is therefore described as a self‐selected volunteer subsample of the national survey cohort rather than a purposively selected sample.

Six interview participants had donated tissue, one was uncertain whether donation had been discussed, and one described a missed opportunity to donate. Participants had experience across chordoma, chondrosarcoma, osteosarcoma and other rare craniospinal tumour pathways. The analysis is presented as contextual, interpretive insight rather than a claim to population‐level representativeness or prevalence.

Sampling adequacy was considered in relation to the focused qualitative aim of the study. Rather than seeking to demonstrate thematic saturation, which Braun and Clarke argue is not fully aligned with reflexive thematic analysis [16], adequacy was judged by the richness, specificity and relevance of the accounts to the study question. The self‐selected design may have favoured participants who were more willing to discuss research or more positively disposed towards tissue donation; this is treated as a limitation on the scope of inference rather than resolved through claims of representativeness.

### 2.3 Interview Procedure and Interview Schedule

Potential interview participants who had opted in through the survey were contacted by the lead researcher and provided with further study information. For this remote interview phase, the ethics‐approved study procedure used documented oral consent supported by prior written study information; participants were not required to return a separate signed consent form. Participants had previously received the Participant Information Sheet and had opted in to further contact after completing the survey.

At the start of each interview, the researcher worked through the approved oral‐consent script, covering study purpose, voluntariness, recording, confidentiality, withdrawal and permission to use anonymised quotations. Consent was documented by the researcher using the approved record‐of‐oral‐consent form before recording began.

A standardised semi‐structured interview guide was used to support consistency while allowing participants to describe their experiences in their own terms. The guide was developed following analysis of the preceding survey and explored selected survey findings in greater experiential depth. Survey findings concerning the circumstances and location of consent, adequacy of time and information, and preferences for advance information and digital support informed corresponding open‐ended questions. The guide explored participants' treatment journeys, motivations for donation, factors that might have altered their decisions, where consent occurred and whether the setting was appropriate, whether sufficient time and information had been provided, the potential value of receiving information in advance, and views on a proposed digital information platform. The complete guide is provided as Supplementary File 3.

Interviews were conducted online by the lead researcher, lasted approximately 45–60 min, were audio‐recorded with consent and professionally transcribed verbatim. Transcripts were anonymised before analysis, and participant identifiers were replaced with study IDs.

### 2.4 Data Analysis

Data were analysed using Braun and Clarke's reflexive thematic analysis approach [17, 18]. Analysis proceeded iteratively through familiarisation, coding, candidate theme development, theme review, defining and naming themes and analytic writing. Data were managed using an Excel‐based coding framework as an organisational aid.

Coding and initial theme development were led by the lead researcher across the complete dataset. A second researcher reviewed selected coded extracts and the developing codebook, considered candidate themes and contributed to analytic meetings in which interpretations and thematic boundaries were challenged and refined. The wider supervisory team reviewed the developing thematic structure and contributed to discussion of the interpretation and final themes. Independent duplicate coding and inter‐coder reliability assessment were not undertaken, consistent with the reflexive thematic analysis approach.

Initial codes were reviewed for patterns of shared meaning, clustered into focused codes and subthemes, and refined into final analytic themes. The development from initial codes to final themes is provided in Supplementary File 1.

### 2.5 Reflexivity and Researcher Positioning

The lead researcher was a nurse‐researcher working in specialist craniospinal tumour care. This provided contextual understanding of clinical pathways and tissue acquisition but also created a potential source of influence. Reflexive memos were used to examine assumptions about the value of donation, research participation and digital consent support, and the analysis actively attended to accounts of uncertainty, non‐recall, preference for paper information, concern about pressure and missed opportunity.

Particular attention was paid to statements of trust in clinical teams because participants may have perceived professional alignment between the interviewer and specialist services. The interviewer's clinical positioning may therefore have contributed to social‐desirability effects or reduced the expression of criticism, and this is considered explicitly in the study limitations.

### 2.6 Ethics

The study was reviewed and approved by the Medical Sciences Interdivisional Research Ethics Committee (MS IDREC), Medical Sciences Division, University of Oxford, UK (R79248/RE001). All participants provided informed consent to participate and for anonymised quotations to be used in publications. For the interview phase, consent was obtained orally and documented using the ethics‐approved procedure described above.

The privacy rights of participants were observed. Transcripts were anonymised, and names, locations and identifiable clinical details were removed or replaced with neutral descriptors. No identifiable patient information is reported.

### 2.7 Patient and Public Contribution

Patient and public involvement informed the development of the wider study pathway. Individuals with lived experience of a primary craniospinal tumour reviewed and provided feedback on the themes and questions developed for the national online survey, and six volunteers with lived experience tested the survey content, language and tone before electronic launch.

The interview phase recruited from survey respondents who actively opted in to further contact and wished to share more detailed accounts of tissue‐donation consent, missed opportunity, information needs and digital support. Patients and members of the public contributed to survey development and testing, but interview participants were not co‐analysts. Their contribution during this phase was as participants whose accounts directly shaped the consent‐support implications.

Patient and public contribution is reported with reference to GRIPP2 short‐form principles, which are intended to improve the transparency and consistency of PPI reporting when involvement is a secondary rather than primary focus [19].

## 3 Results

Four main themes were generated, with an overarching analytic thread describing how participants made sense of tissue donation across the rare tumour pathway. Information support was integrated across the four themes rather than retained as a separate theme. This reflected participants' accounts that information support was not an isolated preference for digital delivery, but part of how donation was understood, revisited, trusted and shared over time.

**Figure 1:**
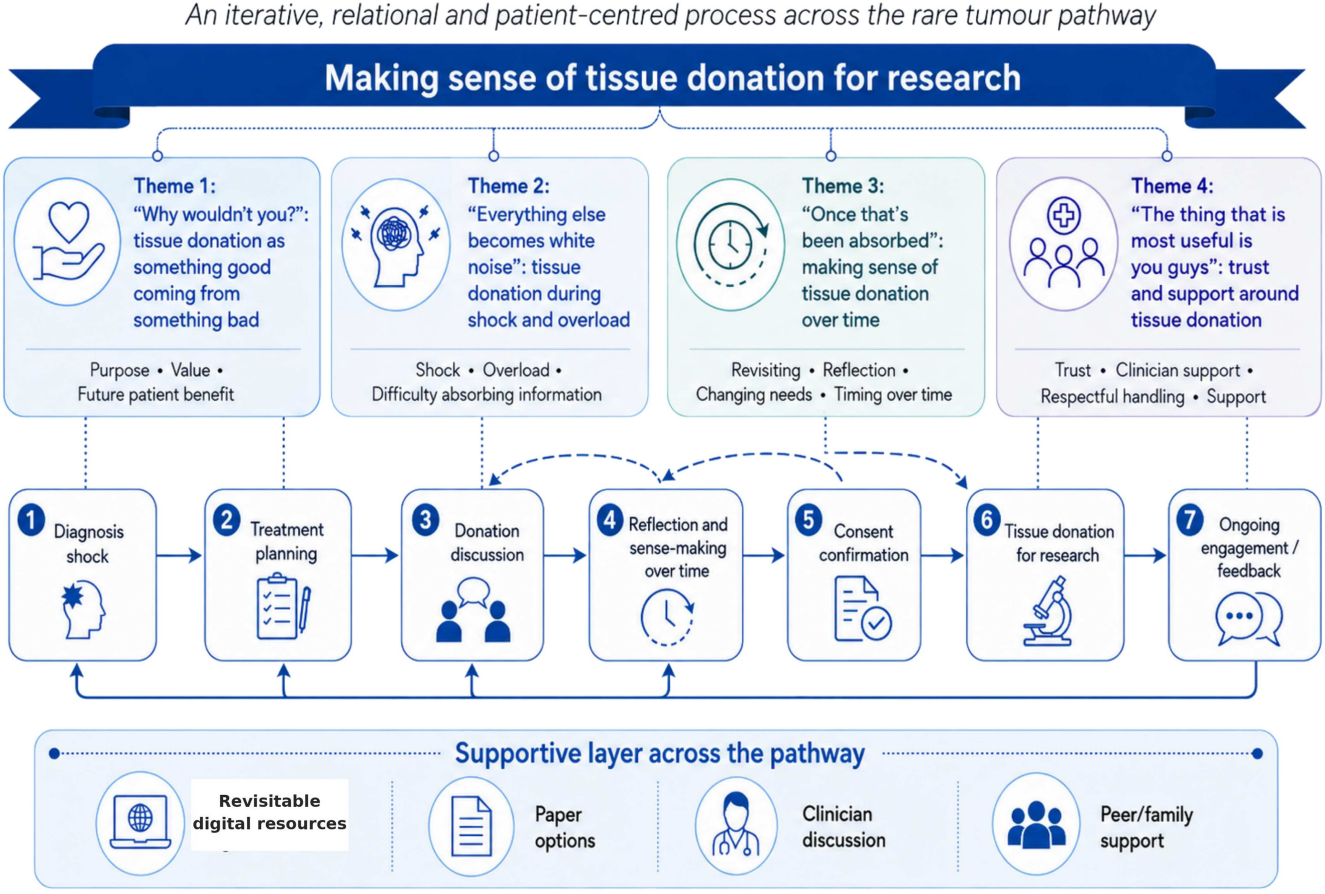
Pathway‐based model of tissue‐donation consent. The model presents tissue‐donation consent as an iterative process across the rare tumour pathway and reflects how participants made sense of donation over time. A supporting layer of digital resources, paper options, clinician discussion and peer/family support is positioned across the pathway to reflect participants' accounts of consent as relational, staged and patient‐centred. Dashed bidirectional arrows between Stages 3 and 5 indicate that donation discussion, reflection and consent confirmation may be revisited iteratively rather than occurring as a fixed linear sequence. The model is a patient‐informed design hypothesis derived from this qualitative sample and has not yet been validated as a clinical pathway.

Eight participants were interviewed: six had donated tissue, one was uncertain whether donation had been discussed, and one reported a missed opportunity to donate. Participants had experience across rare chordoma, chondrosarcoma, osteosarcoma and related craniospinal tumour pathways. To reduce the risk of deductive disclosure in this rare‐disease sample, individual tumour site and donation‐status descriptors are not linked to participant IDs. Themes, subthemes and illustrative quotations are summarised in Table 1; thematic development is provided in Supplementary File 1; and the pathway‐based model is shown in Figure 1.

**Table 1:** Making sense of tissue donation across the rare tumour pathway: themes, subthemes and illustrative quotations.

Theme	Subtheme	Illustrative quotation
‘Why wouldn't you?’: tissue donation as something good coming from something bad	Donation as obvious	‘I just think it's a bit of a no‐brainer, I don't know why you wouldn't’. PIC008
	Helping future patients	‘If whatever it is that I have gone through could possibly in any way help people of the future then brilliant’. PIC001
	Rarity increases value	‘I'd never heard of chordoma before I had it… there's not a huge amount known about it so I felt like it would be valuable’. PIC002
‘Everything else becomes white noise’: tissue donation during shock and overload	Diagnosis shock	‘Anything else that comes along is just white noise’. PIC002
	Surgery‐day anxiety	‘I don't think I'd want to be asked the day of surgery because I was like losing my mind with anxiety and worry’. PIC007
	Unclear what was signed	‘You end up not being sure what you've actually agreed to because you've signed so many bits of paper’. PIC002
‘Once that's been absorbed’: making sense of tissue donation over time	Avoiding pressure	‘Am I being pressured to have surgery in order to get this?’ PIC003
	Missed opportunity	‘I was never, I had many chances to get offered and was never offered’. PIC005
	Changing needs	‘I have noticed that I look for different things at different times’. PIC007
‘The thing that is most useful is you guys’: trust and support around tissue donation	Respectful handling	‘It's not being used as a medical football, my tissue’. PIC004
	Digital access	‘Having a link would probably be good that you just email to someone’. PIC002
	Peer support	‘How frightened should I be?’ PIC003

*Note:* Quotations have been lightly edited only to remove identifying information and improve readability where necessary. The overarching analytic thread, ‘Making sense of tissue donation across the rare tumour pathway’, is woven through all four themes.

### 3.1 Overarching Thread: Making Sense of Tissue Donation Across the Rare Tumour Pathway

Across the dataset, participants described tissue donation as something they made sense of over time, rather than as a single isolated decision. Donation was interpreted through the experience of rare tumour diagnosis, major treatment, uncertainty, trust in specialist teams, family involvement and hope that difficult experiences might benefit future patients. Participants valued information they could return to when ready, share with family and trust as clinically endorsed rather than found through unsupported internet searching.

### 3.2 Theme 1: ‘Why Wouldn't You?’: Tissue Donation as Something Good Coming From Something Bad

Participants commonly described tissue donation as meaningful, worthwhile and morally intuitive. Donation was framed as a way of helping future patients, contributing to research, improving diagnosis and turning a difficult illness experience into something useful. For several participants, the rarity of their tumour increased the perceived value of donation.

I just think it's a bit of a no‐brainer, I don't know why you wouldn't.

(PIC008)

Would you be prepared to let us take some of the tissue for analysis and research? And I said, ‘Why wouldn't I?’

(PIC004)

If whatever it is that I have gone through could possibly in any way help people of the future then brilliant.

(PIC001)

Rarity increased the moral logic of donation. PIC002 had never heard of chordoma before diagnosis and, after discovering how little was known, felt that donating tissue ‘would be valuable’.

PIC003 similarly described rare tumour tissue as a resource through which ‘scientists were getting advanced knowledge’. Donation was also understood pragmatically as making use of tissue already being removed during treatment.

If it isn't coming back to me, why not let somebody make good use of it.

(PIC003)

For several participants, donation was experienced as a meaningful and pragmatic use of tissue already being removed. These accounts also show why a positive attitude towards donation should be interpreted separately from the quality of the consent process, which could still be shaped by timing, voluntariness and trust.

### 3.3 Theme 2: ‘Everything Else Becomes White Noise’: Tissue Donation During Shock and Overload

Participants described diagnosis, treatment planning and surgery as periods of intense emotional and cognitive burden. Their accounts suggested that this context could shape how information was heard, retained or avoided. Whether information had been provided was therefore only one aspect of the consent experience; participants also described whether they felt able to process it at that point in the pathway.

When people tell you you've got cancer, you switch off to most of the rest of the information that you're given. You want to hear how it's going to be treated and anything else that comes along is just white noise.

(PIC002)

By which time I'd gone completely deaf. I couldn't hear a thing.

(PIC004)

There was an awful lot that I must admit that I closed my ears off to. That was my way of dealing with it.

(PIC001)

I'll be completely honest, I don't believe I looked at it. Not because it wasn't of interest, just because I wasn't in a place.

(PIC002)

Recall was also blurred in some accounts. PIC002 described ‘consenting for lots of things’ during a period that felt ‘a little bit of a blur’, while PIC006 could not recall specifically whether tissue donation had been discussed. These accounts illustrate how tissue‐donation discussions can sit within emotionally dense clinical encounters where diagnosis and surgery may narrow attention and make additional information difficult to retain.

I don't think I'd want to be asked the day of surgery because I was like losing my mind with anxiety and worry.

(PIC007)

Participants' support for digital and returnable resources reflected recognition that information delivered during diagnosis and treatment planning was not always absorbable. Leaflets could be lost, ignored or inaccessible during shock and anxiety; alternative formats responded to these pathway realities rather than simple format preference.

### 3.4 Theme 3: ‘Once That's Been Absorbed’: Making Sense of Tissue Donation Over Time

Participants' accounts suggested that acceptability was shaped not only by what was asked, but when and how it was asked. Timing preferences were heterogeneous. Several accounts favoured separation from the initial diagnosis or treatment decision, while one participant found same‐day discussion acceptable. Across these differences, participants emphasised voluntariness and the value of having time or opportunity to revisit information.

The time to discuss tissue donation for research is probably when the patient has consented to say I'm going to have surgery… because the decision has been made by them.

(PIC003)

Discussing that prior to that, you're putting them in a position of what do I do here. Am I being pressured to have surgery in order to get this?

(PIC003)

A subsequent meeting after the original diagnosis when that's been absorbed and… there's a plan in place.

(PIC007)

PIC004 provided an important divergent account: the day of surgery had been acceptable for this participant because they were focused and listening, although he immediately added that this ‘might not be for everybody’. This divergence cautions against treating any single time point as universally optimal and supports flexibility according to patient readiness and clinical context.

Two individual accounts highlighted potential pathway vulnerabilities rather than their frequency. PIC005 described a missed opportunity to donate despite willingness and had assumed removed tissue would automatically be available for research; PIC006 was uncertain whether donation had been discussed. These cases cannot establish how often missed or poorly recalled opportunities occur, but they identify issues that warrant attention in pathway design and future evaluation.

I was never, I had many chances to get offered and was never offered.

(PIC005)

There was an assumption… that yes, it would be used and studied and done for research and I didn't realise that I needed to provide consent for all of that.

(PIC005)

I have noticed that I look for different things at different times.

(PIC007)

Taken together, these accounts suggest potential value in staged and revisitable consent support. Optional digital or paper resources could allow patients to return to information when new questions arise, check what they agreed to and understand how tissue may be used after the initial clinical encounter.

### 3.5 Theme 4: ‘The Thing That Is Most Useful Is You Guys’: Trust and Support Around Tissue Donation

Trust was prominent across the interviews. Participants commonly described confidence in clinical teams and research systems, while also recognising why others might hesitate. Concerns included historic misuse, cultural or religious reasons, uncertainty about where tissue goes, and the distinction between diagnostic, clinical, genomic, biobank and research use. These trust‐related findings should be interpreted alongside the lead interviewer's specialist clinical role, which may have influenced how comfortable participants felt expressing criticism.

There's always stuff in the press, bad news stories and things being mistreated or misused and so I guess I could understand why someone else might feel uncomfortable about that.

(PIC002)

It's not being used as a medical football, my tissue. It's not being kicked around.

(PIC004)

Participants did not all relate to tumour tissue in the same way. PIC002 saw tumour as ‘bits of tissue rather than part of me’ but also acknowledged that ‘other people see that differently’. Consent resources may therefore need to normalise both agreement and refusal and provide proportionate explanations of what tissue donation means, how tissue is handled, why consent is needed and what future use may involve.

You end up not being sure what you've actually agreed to because you've signed so many bits of paper.

— PIC002

Some participants described limitations of paper‐only information and valued clinician‐endorsed digital information as a way to revisit material after the consultation or avoid unsupported internet searching. Digital resources were not described as a replacement for clinician‐led discussion; preferences varied, including an explicit preference to retain a paper option.

Bits of paper get lost and you get given all these leaflets and things and you just put them in your bag and then you find them six months later.

(PIC002)

Having a link would probably be good that you just email to someone.

(PIC002)

A video or… a pictogram, probably something I'd watch… to gain better information. (PIC008)

I would say there should always be a paper option.

(PIC003)

Participants wanted trusted information rather than unsupported internet searching. PIC004 was ‘anti Googling’ because early search results were experienced as ‘horror stories’. PIC008 preferred information from the clinical team and described a digital resource as valuable if it provided clinically endorsed answers rather than random online material. Digital support was particularly valued because questions often arise outside the clinic, including at night.

You do frequently lie in bed at night thinking I can't sleep or I wish I'd asked that question.

(PIC002)

All the information is useful but the thing that is most useful is you guys.

(PIC008)

Although legal consent is individual, several accounts described sense‐making as relational. Family members could help remember, interpret and process information, while peers offered experiential knowledge that participants did not expect written information or clinicians alone to provide. These accounts support offering relational support where wanted while keeping the consent decision clearly with the individual patient.

The session… was recorded, which I've got a copy of, which was really helpful.

(PIC006)

I probably would of, or [my husband] may have if I wasn't feeling up to it.

(PIC007)

Someone that I could just sit down and have a telephone call with or sit down and have a cup of coffee with and say this is what I'm facing and how frightened should I be.

(PIC003)

It would be lovely to meet somebody who has been through the exact same one… something really similar.

(PIC007)

Participants' accounts therefore suggested a wider consent‐support environment shaped by timing, trusted clinical relationships, family or peer support, written and digital resources, and opportunities for reflection, as illustrated in Figure 1. The model is presented as a patient‐informed design hypothesis arising from this small qualitative sample, rather than as a validated clinical pathway.

## 4 Discussion

### 4.1 Principal Findings

In this small, self‐selected volunteer sample, participants were generally supportive of tissue donation and often framed it as altruistic, pragmatic and future‐facing. At the same time, their accounts described diagnosis shock, information overload, blurred recall, variable timing preferences, questions about governance, relational support and occasional missed or uncertain opportunities. These qualitative findings complement the preceding national survey, which identified generally positive experiences alongside practical opportunities to strengthen consent support through appropriate timing, revisitable information, opportunities for questions and family‐inclusive decision‐making [1].

The finding that high willingness can coexist with incomplete understanding or imperfect consent experiences is established in the wider oncology biobanking literature. The contribution of this study is therefore more specific: it examines how these issues are experienced within rare craniospinal tumour pathways and uses participants' accounts to generate cautious, patient‐informed hypotheses about pathway design, including flexibility in timing, revisitability, trusted information and relational support.

### 4.2 Extending Established Biobanking Findings Into Rare Craniospinal Pathways

Participants' positive orientation towards donation is consistent with wider oncology biobanking research. Braun et al. reported high willingness to donate, including altruistic and pragmatic motivations [2], and Yip et al. described biobanking as a ‘no brainer’ alongside the importance of trust, timing and family or emotional support [3]. Fradgley et al. similarly found that many cancer patients were willing to participate if asked, while clinician‐dependent opt‐in processes could create attrition, missed opportunity and poor recall of consent [5, 6]. Systematic review evidence also shows that several elements of biobanking consent are often poorly understood [4].

The rare craniospinal context nevertheless adds a distinct patient‐experience perspective. Qualitative work with people facing high‐grade brain cancer has shown that post‐mortem brain donation may provide purpose, control and legacy [8], while research involving paediatric high‐grade glioma donation has identified altruism alongside a need for clear procedural information [9]. Rare‐cancer research‐engagement literature also emphasises the importance of patient advocacy, respect, trust, inclusivity and sustained relationships with research teams [10, 11]. These studies are not directly equivalent to tissue donation during adult craniospinal surgery, but they reinforce the importance of understanding donation within its wider clinical and relational context.

The contribution of the present analysis is therefore to shift the emphasis from whether donation is acceptable towards what features of a rare‐tumour pathway may help consent to be experienced as informed, voluntary and appropriately supported. The interviews identify plausible service‐design considerations, but the small, predominantly donation‐positive and self‐selected volunteer sample means that these should be treated as hypotheses for refinement and evaluation rather than as universal recommendations.

### 4.3 Consent as a Relational and Staged Process

Participants' accounts were consistent with a relational and staged view of consent. NICE guidance on shared decision‐making frames decision‐making as a collaborative process supported before, during and after discussions rather than as a single information‐transfer event [20]. NICE also recommends involving family members or supporters where the person wishes, allowing time to decide, offering further opportunities for discussion and providing resources after consultations in a preferred format. Although this guidance concerns healthcare decision‐making rather than tissue donation specifically, it offers a useful comparator for the support described by participants.

UK professional and legal guidance also emphasises clear information, adequate time and attention to factors such as fear, confusion, shock, fatigue or pain that may affect decision‐making [21]. These principles again provide contextual support rather than direct evidence for a specific tissue‐donation pathway.

Participants also described decision‐making and sense‐making as relational. This is compatible with work on relational autonomy, including Gilbar's analysis that family involvement can support informed decisions while leaving the final decision with the patient [22]. In the present sample, family members could help retain information and ask questions; peers offered experiential knowledge; and clinicians were described as important sources of trust. These findings suggest that consent support can acknowledge relationships around the patient without weakening individual voluntariness.

### 4.4 From Participant Insights to Consent‐Support Design

Participants' accounts suggest several candidate design principles: stage information where feasible; make voluntariness explicit; distinguish research consent from treatment decisions; and enable patients to revisit information or check what they agreed to. These ideas are consistent with informed‐consent intervention literature in clinical care and research, where enhanced materials, extended discussion and other structured supports have been evaluated to improve understanding and decision‐making [23, 24].

Evidence from residual‐tissue research provides a further comparator. Rebers et al. found that an ‘opt‐out plus’ procedure combining opt‐out consent with active information provision achieved high tissue availability while supporting patient informedness and healthcare‐provider satisfaction [25]. This reinforces the importance of information provision, but the present qualitative data do not establish one optimal consent model.

Taken together, the interviews support evaluating a layered model of consent support that combines clinician discussion with optional paper and digital resources, including links, videos, diagrams, frequently asked questions, question prompts, consent records, research updates and signposting. Figure 1 should be read as a patient‐informed model for further refinement rather than as a validated intervention.

### 4.5 Implications for Digital Adjuncts

Participants' interest in digital resources should not be interpreted as a preference for replacing clinician‐led consent. Digital adjuncts were valued because they could extend trusted communication beyond the consultation, support family involvement and reduce reliance on unsupported internet searching. A systematic review of digital consent tools found no negative effect across reported outcomes but highlighted heterogeneity and the need for robust evaluation [26]. This also aligns with dynamic‐consent literature describing digital interfaces that can support ongoing engagement and allow participants to revisit information or preferences over time [27, 28].

Dynamic consent has been proposed as a response to static, paper‐based consent in biobanking and data‐intensive biomedical research [27, 29]. The present findings suggest that any digital component should be evaluated as a facilitative adjunct rather than assumed to substitute for relational clinical discussion.

This is particularly important because digital consent strategies can introduce risks of exclusion through the digital divide and require appropriate resources, governance and evaluation [26, 28, 29].

Broader research‐participation evidence also supports attention to equity and patient engagement. A recent meta‐review identified barriers for equity‐deserving groups and highlighted patient engagement and shared decision‐making [30], while rare‐cancer engagement studies emphasise respect, trust, inclusivity, relationships and empowerment [10, 11]. These considerations reinforce the need to evaluate whether any proposed consent‐support pathway is accessible to people who are less digitally confident, less positively disposed towards research or less connected to specialist services.

### 4.6 Implications for Rare Tumour Services and Practice

Table 2 presents participant‐derived implications for consent‐support pathway design. Reflexive thematic analysis does not treat frequency as a proxy for analytic importance; the evidence‐basis column therefore distinguishes recurrent patterns, divergent accounts and single‐case signals rather than presenting quasi‐quantitative prevalence estimates.

**Table 2:** Participant‐derived implications for tissue‐donation consent‐support pathway design.

Practice area	Evidence basis in this data set	Cautious implication for consent‐support design
Timing	Recurrent concern across Themes 2 and 3, with heterogeneous preferences; one participant found same‐day discussion acceptable.	Build flexibility into timing and allow discussion to be revisited rather than applying a single fixed time point.
Voluntariness	An explicit pressure concern arose in one account and was consistent with the broader timing and framing theme.	Clearly distinguish research participation from treatment decisions and reiterate that declining will not affect care.
Opportunity	One participant described a missed opportunity, and one was uncertain whether donation had been discussed.	Use reliable service prompts or processes so that an opportunity to consider donation does not depend solely on patient initiative; the frequency of missed opportunity requires further evaluation.
Information and governance	Several accounts described blurred recall or uncertainty about what had been signed, tissue handling or future use.	Provide proportionate plain‐language information and a way to revisit what was agreed, including relevant governance and future‐use information.
Support format	Participants expressed different preferences for clinician discussion, paper and digital resources.	Offer more than one accessible format, with digital tools as optional adjuncts rather than replacements for clinician discussion or paper alternatives.
Relational support and follow‐up	Family, peer and clinician support appeared across several accounts; interest in later questions and research feedback also emerged.	Where the patient wishes, facilitate supporter involvement and consider ways to revisit questions, consent records or research information after the initial discussion.

### 4.7 Strengths and Limitations

This study provides in‐depth insight from an under‐researched rare tumour population and is strengthened by its integration within a wider mixed‐methods programme, use of direct quotation, reflexive thematic analysis and a transparent codebook and thematic‐development supplement. The dataset includes donated‐tissue experiences alongside uncertain recall, a divergent same‐day timing preference and a single missed‐opportunity account. These variations were retained in the analysis rather than forcing a uniform pathway narrative.

The study also has important limitations. Eight participants formed a self‐selected volunteer subsample of the national survey cohort, six of whom had donated tissue. All 50 survey respondents were invited to volunteer, 13 expressed interest, 8 ultimately provided informed consent, and all 8 were interviewed. No further selection was undertaken by the research team according to diagnosis, donation status, participant characteristics or expressed views. Nevertheless, participant self‐selection and the two‐stage opt‐in process may have preferentially attracted people who were more interested in research, more willing to discuss their experiences or more positively disposed towards tissue donation.

The findings cannot estimate the prevalence of missed opportunities, preferred timing or information needs across craniospinal tumour populations. Single cases are therefore treated as signals of potential pathway vulnerabilities rather than evidence that those experiences are common. The internet‐enabled, English‐language recruitment route may also have under‐represented people experiencing digital exclusion or those with different language and communication needs.

The lead researcher was a specialist clinician in this field. This positioning supported contextually informed interviewing but may also have generated social‐desirability effects, particularly in Theme 4 accounts of trust in clinical teams, or reduced participants' willingness to express criticism. Reflexive memoing, discussion with the wider research team and attention to divergent accounts were used to examine this influence, but it cannot be removed. The findings should therefore be understood as contextual and hypothesis‐generating, with transferability requiring consideration of other patient groups and service settings and further evaluation in larger and more diverse populations.

## 5 Conclusion

Participants in this small, self‐selected volunteer sample commonly framed tissue donation as meaningful and worthwhile, while also describing how timing, emotional overload, recall, trust and support could shape the consent experience. The findings do not establish a single optimal timing or validated pathway. Rather, they suggest that rare craniospinal tumour services could evaluate flexible, staged and revisitable approaches that preserve voluntariness, enable trusted information to be revisited, and offer relational or digital support where wanted. Figure 1 is therefore best understood as a patient‐informed design hypothesis for further refinement and evaluation.

## Author Contributions

**Gerard Mawhinney:** conceptualisation, writing – original draft, methodology, formal analysis, project administration, data curation, software, visualisation, writing – review and editing, validation, investigation, resources. **Simona Fourie:** methodology, writing – review and editing, formal analysis, validation, resources, conceptualisation, data curation. **Simon Leedham:** writing – review and editing, supervision, methodology, conceptualisation, resources. **Helen Higham:** conceptualisation, methodology, writing – review and editing, supervision, resources, validation. **Olaf Ansorge:** funding acquisition, conceptualisation, methodology, supervision, writing – review and editing, resources.

This work is undertaken as part of a University of Oxford DPhil programme. This work was supported by United Kingdom Research and Innovation (UKRI) (grant MR/X004317/1). The funder had no role in the design, conduct, analysis or reporting of the research.

## Conflicts of Interest

The authors declare no conflicts of interest.

## Supporting information


Supporting File 1

Supporting File 2

Supporting File 3

## Data Availability

Due to the sensitive nature of the interview data and the rarity of the participant population, full transcripts are not publicly available. De‐identified excerpts supporting the analysis are presented within the article. Further data‐access requests will be considered in accordance with the study ethics approval and institutional governance requirements.

## References

[1] G. Mawhinney , H. Higham , S. Leedham , and O. Ansorge , “Patient Experiences of Tissue Donation and Digital Consent Support in Primary Craniospinal Tumour Research,” Supportive Care in Cancer 34 (2026): 774, 10.1007/s00520-026-11017-x. 42470522 PMC13380566

[2] K. L. Braun , J. U. Tsark , A. Powers , et al., “Cancer Patient Perceptions About Biobanking and Preferred Timing of Consent,” Biopreservation and Biobanking 12, no. 2 (2014): 106–112, 10.1089/bio.2013.0083. 24749877 PMC3995435

[3] S. Yip , J. Fleming , H. L. Shepherd , A. Walczak , J. Clark , and P. Butow , “‘As Long as You Ask’: A Qualitative Study of Biobanking Consent—Oncology Patients' and Health Care Professionals' Attitudes, Motivations, and Experiences—The B‐PPAE Study,” Oncologist 24, no. 6 (2019): 844–856, 10.1634/theoncologist.2018-0233. 30413662 PMC6656517

[4] E. R. Eisenhauer , A. R. Tait , S. Y. Rieh , and C. M. Arslanian‐Engoren , “Participants' Understanding of Informed Consent for Biobanking: A Systematic Review,” Clinical Nursing Research 28, no. 1 (2019): 30–51, 10.1177/1054773817722690. 28745067

[5] E. A. Fradgley , S. E. Chong , M. E. Cox , C. Gedye , and C. L. Paul , “Patients' Experiences and Preferences for Opt‐in Models and Health Professional Involvement in Biobanking Consent: A Cross‐Sectional Survey of Australian Cancer Outpatients,” Asia‐Pacific Journal of Clinical Oncology 15, no. 1 (2019): 31–37, 10.1111/ajco.12866. 29573159

[6] E. A. Fradgley , S. E. Chong , M. E. Cox , C. L. Paul , and C. Gedye , “Enlisting the Willing: A Study of Healthcare Professional‐Initiated and Opt‐In Biobanking Consent Reveals Improvement Opportunities Throughout the Registration Process,” European Journal of Cancer 89 (2018): 36–41, 10.1016/j.ejca.2017.10.025. 29223480

[7] S. Joffe , E. F. Cook , P. D. Cleary , J. W. Clark , and J. C. Weeks , “Quality of Informed Consent in Cancer Clinical Trials: A Cross‐Sectional Survey,” Lancet 358, no. 9295 (2001): 1772–1777, 10.1016/S0140-6736(01)06805-2. 11734235

[8] C. P. Griffin , M. A. Carlson , M. M. Walker , J. Lynam , and C. L. Paul , “‘It's My Wish, We Need This Program’: Qualitative Reflections From People With High‐Grade Brain Cancer Consenting to Postmortem Brain Donation,” JCO Oncology Practice, ahead of print, November 10, 2025, 10.1200/OP-25-00375. PMC13592821 41213095

[9] E. G. Robertson , C. E. Wakefield , M. Tsoli , et al., “Parents' Experiences of Postmortem Tumor Donation for High‐Grade Gliomas: Benefits and Suggested Improvements,” Neurooncology Advances 3, no. 1 (2021): vdab087, 10.1093/noajnl/vdab087. PMC8386242 34458732

[10] D. K. Reinke , “Meaningful Engagement of the Patient in Rare Cancer Research: Sarcoma as an Exemplar,” Current Problems in Cancer 45, no. 4 (2021): 100772, 10.1016/j.currproblcancer.2021.100772. 34289946

[11] V. Venkataraman , L. Weber , L. Fisher , et al., “Supporting Participant Engagement in Cancer Genomics Research in Rare Cancers: A Qualitative Study of Patients, Caregivers, and Advocates,” Cancer Control 32 (2025): 10732748251364041, 10.1177/10732748251364041. 40719570 PMC12304653

[12] G. Mawhinney , H. Higham , S. Leedham , and O. Ansorge , “Study Protocol for Putting the ‘Person’ in the PiCTuRE: An Exploratory Sequential Mixed Methods‐Based Design, Exploring How Precision Medicine Is Implemented and Experienced by People Living With a Primary Tumour of the Craniospinal Axis,” BMC Cancer 25 (2025): 420, 10.1186/s12885-025-13795-9. 40055688 PMC11889933

[13] L. Varpio , E. Paradis , S. Uijtdehaage , and M. Young , “The Distinctions Between Theory, Theoretical Framework, and Conceptual Framework,” Academic Medicine 95, no. 7 (2020): 989–994, 10.1097/ACM.0000000000003075. 31725464

[14] M. Bearman , “Focus on Methodology: Eliciting Rich Data—A Practical Approach to Writing Semi‐Structured Interview Schedules,” Focus on Health Professional Education 20, no. 3 (2019): 1–11, 10.11157/fohpe.v20i3.387.

[15] A. Tong , P. Sainsbury , and J. Craig , “Consolidated Criteria for Reporting Qualitative Research (COREQ): A 32‐Item Checklist for Interviews and Focus Groups,” International Journal for Quality in Health Care 19, no. 6 (2007): 349–357, 10.1093/intqhc/mzm042. 17872937

[16] V. Braun and V. Clarke , “To Saturate or Not to Saturate? Questioning Data Saturation as a Useful Concept for Thematic Analysis and Sample‐Size Rationales,” Qualitative Research in Sport, Exercise and Health 13, no. 2 (2021): 201–216, 10.1080/2159676X.2019.1704846.

[17] V. Braun and V. Clarke , “Using Thematic Analysis in Psychology,” Qualitative Research in Psychology 3, no. 2 (2006): 77–101, 10.1191/1478088706qp063oa.

[18] V. Braun and V. Clarke , “Toward Good Practice in Thematic Analysis: Avoiding Common Problems and Be(com)ing a Knowing Researcher,” International Journal of Transgender Health 24, no. 1 (2023): 1–6, 10.1080/26895269.2022.2129597. 36713144 PMC9879167

[19] S. Staniszewska , J. Brett , I. Simera , et al., “GRIPP2 Reporting Checklists: Tools to Improve Reporting of Patient and Public Involvement in Research,” Research Involvement and Engagement 3 (2017): 13, 10.1186/s40900-017-0062-2. 29062538 PMC5611595

[20] National Institute for Health and Care Excellence , “Shared Decision Making,” NICE Guideline NG197, Published June 17, 2021, https://www.nice.org.uk/guidance/ng197. 34339147

[21] General Medical Council , “Key Legislation and Case Law Relating to Decision Making and Consent,” Published 2020, https://www.gmc-uk.org/-/media/gmc-site/ethical-guidance/related-pdf-items/factsheet---key-legislation-and-case-law-relating-to-decision-making-and-consent-84176182.pdf.

[22] R. Gilbar , “Family Involvement, Independence, and Patient Autonomy in Practice,” Medical Law Review 19, no. 2 (2011): 192–234, 10.1093/medlaw/fwr008. 21543361

[23] P. Kinnersley , K. Phillips , K. Savage , et al., “Interventions to Promote Informed Consent for Patients Undergoing Surgical and Other Invasive Healthcare Procedures,” Cochrane Database of Systematic Reviews 2013, no. 7 (2013): CD009445, 10.1002/14651858.CD009445.pub2. 23832767 PMC11663509

[24] A. Nishimura , J. Carey , P. J. Erwin , J. C. Tilburt , M. H. Murad , and J. B. McCormick , “Improving Understanding in the Research Informed Consent Process: A Systematic Review of 54 Interventions Tested in Randomized Control Trials,” BMC Medical Ethics 14 (2013): 28, 10.1186/1472-6939-14-28. 23879694 PMC3733934

[25] S. Rebers , E. Vermeulen , A. P. Brandenburg , et al., “A Randomised Controlled Trial of Consent Procedures for the Use of Residual Tissues for Medical Research: Preferences of and Implications for Patients, Research, and Clinical Practice,” PLoS One 11, no. 3 (2016): e0152509, 10.1371/journal.pone.0152509. 27028128 PMC4814081

[26] F. Gesualdo , M. Daverio , L. Palazzani , et al., “Digital Tools in the Informed Consent Process: A Systematic Review,” BMC Medical Ethics 22 (2021): 18, 10.1186/s12910-021-00585-8. 33639926 PMC7913441

[27] J. Kaye , E. A. Whitley , D. Lund , M. Morrison , H. Teare , and K. Melham , “Dynamic Consent: A Patient Interface for Twenty‐First Century Research Networks,” European Journal of Human Genetics 23, no. 2 (2015): 141–146, 10.1038/ejhg.2014.71. 24801761 PMC4130658

[28] H. J. A. Teare , M. Prictor , and J. Kaye , “Reflections on Dynamic Consent in Biomedical Research: The Story so Far,” European Journal of Human Genetics 29, no. 4 (2021): 649–656, 10.1038/s41431-020-00771-z. 33249421 PMC7695991

[29] L. Goncharov , H. Suominen , and M. Cook , “Dynamic Consent and Personalised Medicine,” Medical Journal of Australia 216, no. 11 (2022): 547–549, 10.5694/mja2.51555. 35611469 PMC9544476

[30] T. L. Morgan , K. Carroll , A. Waqar , et al., “A Meta‐Review of Patient Engagement, Shared Decision‐Making, and Factors Influencing Equity‐Deserving Populations' Participation in Clinical Trials,” Research Involvement and Engagement 12 (2026): 50, 10.1186/s40900-026-00868-7. 41840453 PMC13104246

