# Supplementary material for: ‘A No‐Brainer, but Not a Simple Decision’: A Qualitative Study of Patient Experiences of Tissue Donation for Research in Rare Craniospinal Tumour Pathways
Source: Health Expect. 2026 Aug 22;29(4):e70839. doi: 10.1111/hex.70839 (PMC13499010; doi:10.1111/hex.70839)
Supplement: Supplementary file 1 [file HEX-29-e70839-s001.docx]

**Supplementary File 1. Codebook and thematic development**

**Table 1. Development of themes from initial codes to final analytic themes**

| **Initial codes** | **Focused codes / subthemes** | **Final analytic theme** | **Illustrative quotations** |
| --- | --- | --- | --- |
| helping future patients; turning bad into good; it is coming out anyway; no-brainer; why wouldn’t I; research contribution; rare tumour value; future generations; diagnosis improvement; knowledge generation; research updates; connection to future patient benefit | altruistic contribution; rarity as value amplifier; pragmatic use of unwanted tissue; future diagnosis and treatment benefit; digital resources as a way of maintaining connection to purpose and impact | Overarching analytic thread: Making sense of tissue donation across the rare tumour pathway  Theme 1: “Why wouldn’t you?”: tissue donation as something good coming from something bad  Donation was constructed as meaningful, altruistic and pragmatic, particularly when tissue was already being removed and could contribute to rare-tumour knowledge. | PIC008: “I just think it’s a bit of a no-brainer, I don’t know why you wouldn’t.” PIC004: “Would you be prepared to let us take some of the tissue for analysis and research? And I said, ‘Why wouldn’t I?’” |
| white noise; switching off; closed ears; blurred recall; too many forms; not in a place to read; diagnosis shock; surgery-day anxiety; fear; information overload; leaflets lost; not absorbable in the moment | diagnosis shock and reduced processing; information avoidance as coping; blurred recall and multiple consent decisions; surgery-day anxiety; need for revisitable information after emotional overload | Theme 2: “Everything else becomes white noise”: tissue donation during shock and overload  Consent occurred within diagnosis shock, surgical anxiety and information burden, affecting attention, recall and readiness to engage with research information. | PIC002: “When people tell you you’ve got cancer, you switch off to most of the rest of the information… anything else that comes along is just white noise.” PIC004: “By which time I’d gone completely deaf. I couldn’t hear a thing.” |
| right time to ask; after surgery decision; avoid pressure; not diagnosis day; not surgery day; pre-op check-in; same-day acceptable for some; opening the door; missed opportunity; not asked; assumed tissue would be used; changing needs over time; questions emerging later | pathway-based timing; voluntariness and avoiding perceived coercion; heterogeneous timing preferences; missed opportunities and patient assumptions; tissue donation as an evolving rather than single decision point | Theme 3: “Once that’s been absorbed”: making sense of tissue donation over time  Acceptability depended on when and how donation was introduced, whether voluntariness was clear, and whether the pathway reliably created an opportunity to donate and revisit information. | PIC003: “The time to discuss tissue donation for research is probably when the patient has consented to say I’m going to have surgery… because the decision has been made by them.” PIC007: “I have noticed that I look for different things at different times.” |
| bad press; tissue misuse; medical football; body/tissue as personal; others see tissue differently; what did I sign?; biobank/future use; research feedback; governance clarity; legitimise refusal; avoid Google; trusted information; clinician relationship; family access; peer support; recorded consultation; rare tumour isolation | trust and historical concerns; respectful handling of tissue; clarity about what was signed; feedback about research impact; digital as trusted alternative to open internet searching; family, peer and clinician support around consent | Theme 4: “The thing that is most useful is you guys”: trust and support around tissue donation  Participants trusted clinical teams but wanted clear, proportionate information about tissue handling, consent requirements, future use and research impact. Consent and information needs extended beyond the individual patient to include family, supporters, peers and clinician relationships. | PIC004: “It’s not being used as a medical football, my tissue.” PIC008: “I didn’t want to Google it… I’d rather just hear it from you guys.” PIC003: “How frightened should I be?” |

**Table 2. Manuscript theme summary with quote anchors**

| **Final theme** | **Subtheme / focused code** | **Participant** | **Quote anchor** |
| --- | --- | --- | --- |
| Overarching analytic thread: Making sense of tissue donation across the rare tumour pathway | Overarching sense-making across diagnosis, treatment, donation, trust and support | PIC002 | “I have noticed that I look for different things at different times.” |
| Theme 1: “Why wouldn’t you?”: tissue donation as something good coming from something bad | Donation as obvious | PIC008 | “I just think it’s a bit of a no-brainer, I don’t know why you wouldn’t.” |
| Theme 1: “Why wouldn’t you?”: tissue donation as something good coming from something bad | Donation as obvious | PIC004 | “Would you be prepared to let us take some of the tissue for analysis and research? And I said, ‘Why wouldn’t I?’” |
| Theme 1: “Why wouldn’t you?”: tissue donation as something good coming from something bad | Helping future patients | PIC001 | “If whatever it is that I have gone through could possibly in any way help people of the future then brilliant.” |
| Theme 1: “Why wouldn’t you?”: tissue donation as something good coming from something bad | Rarity increases value | PIC002 | “I’d never heard of chordoma before I had it… there’s not a huge amount known about it so I felt like it would be valuable.” |
| Theme 1: “Why wouldn’t you?”: tissue donation as something good coming from something bad | Pragmatic use of removed tissue | PIC003 | “If it isn’t coming back to me, why not let somebody make good use of it.” |
| Theme 2: “Everything else becomes white noise”: tissue donation during shock and overload | Diagnosis shock / white noise | PIC002 | “When people tell you you’ve got cancer, you switch off to most of the rest of the information… anything else that comes along is just white noise.” |
| Theme 2: “Everything else becomes white noise”: tissue donation during shock and overload | Information shutdown | PIC004 | “By which time I’d gone completely deaf. I couldn’t hear a thing.” |
| Theme 2: “Everything else becomes white noise”: tissue donation during shock and overload | Selective avoidance as coping | PIC001 | “There was an awful lot that I must admit that I closed my ears off to. That was my way of dealing with it.” |
| Theme 2: “Everything else becomes white noise”: tissue donation during shock and overload | Not ready to read information | PIC002 | “I don’t believe I looked at it. Not because it wasn’t of interest, just because I wasn’t in a place.” |
| Theme 2: “Everything else becomes white noise”: tissue donation during shock and overload | Surgery-day anxiety | PIC007 | “I don’t think I’d want to be asked the day of surgery because I was like losing my mind with anxiety and worry.” |
| Theme 3: “Once that’s been absorbed”: making sense of tissue donation over time | After treatment decision | PIC003 | “The time to discuss tissue donation for research is probably when the patient has consented to say I’m going to have surgery… because the decision has been made by them.” |
| Theme 3: “Once that’s been absorbed”: making sense of tissue donation over time | Avoiding perceived pressure | PIC003 | “Am I being pressured to have surgery in order to get this?” |
| Theme 3: “Once that’s been absorbed”: making sense of tissue donation over time | Subsequent/pre-operative meeting | PIC007 | “A subsequent meeting after the original diagnosis when that’s been absorbed and… there’s a plan in place.” |
| Theme 3: “Once that’s been absorbed”: making sense of tissue donation over time | Missed opportunity | PIC005 | “I was never, I had many chances to get offered and was never offered.” |
| Theme 3: “Once that’s been absorbed”: making sense of tissue donation over time | Consent assumption | PIC005 | “There was an assumption… that yes, it would be used and studied and done for research and I didn’t realise that I needed to provide consent for all of that.” |
| Theme 3: “Once that’s been absorbed”: making sense of tissue donation over time | Changing needs over time | PIC007 | “I have noticed that I look for different things at different times.” |
| Theme 4: “The thing that is most useful is you guys”: trust and support around tissue donation | Respectful tissue handling | PIC004 | “It’s not being used as a medical football, my tissue.” |
| Theme 4: “The thing that is most useful is you guys”: trust and support around tissue donation | Unclear what was signed | PIC002 | “You end up not being sure what you’ve actually agreed to because you’ve signed so many bits of paper.” |
| Theme 4: “The thing that is most useful is you guys”: trust and support around tissue donation | Digital link | PIC002 | “Having a link would probably be good that you just email to someone.” |
| Theme 4: “The thing that is most useful is you guys”: trust and support around tissue donation | Avoiding Google | PIC004 | “I am very much anti Googling stuff because for the first 10 pages it’s all horror stories.” |
| Theme 4: “The thing that is most useful is you guys”: trust and support around tissue donation | Clinician-trusted information | PIC008 | “I didn’t want to Google it… I’d rather just hear it from you guys.” |
| Theme 4: “The thing that is most useful is you guys”: trust and support around tissue donation | Recorded consultation / family sharing | PIC006 | “The session… was recorded, which I’ve got a copy of, which was really helpful.” |
| Theme 4: “The thing that is most useful is you guys”: trust and support around tissue donation | Family access when patient not ready | PIC007 | “I probably would of, or [my husband] may have if I wasn’t feeling up to it.” |
| Theme 4: “The thing that is most useful is you guys”: trust and support around tissue donation | Peer support need | PIC003 | “Someone that I could just sit down and have a telephone call with… and say this is what I’m facing and how frightened should I be.” |

**Table 3. Supportive layers across the pathway: support sought during participants’ experiences**

| **Support component** | **What participants sought** | **Analytic function** | **Quote anchors** |
| --- | --- | --- | --- |
| Digital resources that can be returned to | Email/web links, videos, pictograms, FAQs, question prompts, consent records and research updates that could be returned to after the clinical encounter. | Supported delayed sense-making, family sharing, recall and trusted access to information outside clinic. | PIC002: “Having a link would probably be good that you just email to someone.” PIC002: “You do frequently lie in bed at night thinking I can’t sleep or I wish I’d asked that question.” PIC008: “A video or… a pictogram, probably something I’d watch… to gain better information.” |
| Paper options | A paper alternative alongside digital information. | Protected accessibility, preference and choice; avoided positioning digital resources as the only acceptable route. | PIC003: “I would say there should always be a paper option.” PIC007: “Some people might want a leaflet. Some people might want to go online. Some people just might not want to know.” |
| Clinician discussion | Clinician-endorsed information, opportunity to ask questions and reassurance that information was safe, accurate and relevant. | Provided trust, credibility and emotional containment; reduced reliance on unsupported internet searching. | PIC008: “All the information is useful but the thing that is most useful is you guys.” PIC008: “I didn’t want to Google it… I’d rather just hear it from you guys.” |
| Peer/family support | Recorded consultations, family-accessible information, partner involvement and contact with someone with lived experience. | Recognised consent as relationally supported: family and peers helped participants remember, interpret and make sense of tissue donation. | PIC006: “The session… was recorded, which I’ve got a copy of, which was really helpful.” PIC007: “I probably would of, or [my husband] may have if I wasn’t feeling up to it.” PIC003: “How frightened should I be?” |

Note. The supportive layer is presented as an overall observation across the four themes. It summarises support sought during participants’ experiences rather than constituting an additional theme.
