# Supplementary material for: ‘A No‐Brainer, but Not a Simple Decision’: A Qualitative Study of Patient Experiences of Tissue Donation for Research in Rare Craniospinal Tumour Pathways
Source: Health Expect. 2026 Aug 22;29(4):e70839. doi: 10.1111/hex.70839 (PMC13499010; doi:10.1111/hex.70839)
Supplement: Supplementary file 2 [file HEX-29-e70839-s002.docx]

**Supplementary File 2. Completed COREQ Checklist**

Consolidated Criteria for Reporting Qualitative Research (COREQ): 32-item checklist for interviews and focus groups.

| **Domain/section** | **Item** | **Guide question** | **Response for this manuscript** |
| --- | --- | --- | --- |
| **Domain 1: Research team and reflexivity** |  |  |  |
| Personal characteristics | 1 | Interviewer/facilitator: Which author/s conducted the interview? | The lead researcher conducted the semi-structured interviews. |
|  | 2 | Credentials: What were the researcher’s credentials? | The interviewer was a registered nurse, consultant-level clinician and doctoral researcher with postgraduate qualitative research training. |
|  | 3 | Occupation: What was their occupation at the time of the study? | The interviewer was working in specialist craniospinal tumour care and was undertaking doctoral research at the time of the study. |
|  | 4 | Gender: Was the researcher male or female? | Male |
|  | 5 | Experience and training: What experience or training did the researcher have? | The interviewer was an experienced clinician-researcher working in specialist craniospinal tumour care, with doctoral-level qualitative research training and supervision. Reflexive thematic analysis was supported by reflexive memoing and supervisory discussion. |
| Relationship with participants | 6 | Relationship established: Was a relationship established prior to study commencement? | Participants were introduced to the interviewer at recruitment/interview and were informed of the researcher role and study purpose. The manuscript reports the interviewer’s clinician-researcher position and reflexive handling of this role. |
|  | 7 | Participant knowledge of the interviewer: What did participants know about the researcher? | Participants knew the interviewer’s name, university affiliation, clinician-researcher role and that the study formed part of a doctoral research programme on tissue-donation consent in craniospinal tumour pathways. |
|  | 8 | Interviewer characteristics: What characteristics were reported about the interviewer/facilitator? | The manuscript reports that the lead researcher was a clinician-researcher working in specialist craniospinal tumour care. Reflexivity addressed how clinical familiarity with donation, research participation and digital consent support could shape interpretation. |
| **Domain 2: Study design** |  |  |  |
| Theoretical framework | 9 | Methodological orientation and theory: What methodological orientation was stated to underpin the study? | A qualitative interview study with an experiential and interpretive orientation. Data were analysed using Braun and Clarke’s reflexive thematic analysis. |
| Participant selection | 10 | Sampling: How were participants selected? | Purposive sampling was used. Interview participants were recruited from respondents to the preceding national online survey who actively opted in to be contacted for a further interview by selecting the interview option at the end of the survey and providing an email address. |
|  | 11 | Method of approach: How were participants approached? | Participants first completed the national online survey. At the end of the survey, respondents were invited to volunteer for an additional short online interview; those willing to be contacted selected the opt-in box and provided an email address. The lead researcher then contacted potential participants with further study information and arranged the interview. Separate consent was taken for the interview phase. |
|  | 12 | Sample size: How many participants were in the study? | Eight participants took part in qualitative interviews. Fifty participants completed the preceding online survey; thirteen survey respondents expressed interest in being contacted for interview and eight subsequently agreed to participate. |
|  | 13 | Non-participation: How many people refused to participate or dropped out? Reasons? | Thirteen survey respondents expressed interest in interview contact; eight completed interviews. Five did not proceed to interview. No reasons were provided by individuals who did not proceed after initially expressing interest; however, non-participation may have reflected ongoing treatment demands, changes in health or personal circumstances, or a subsequent decision not to participate. |
| Setting | 14 | Setting of data collection: Where was the data collected? | Interviews were conducted online; participants joined from a location of their choice. |
|  | 15 | Presence of non-participants: Was anyone else present besides participants and researchers? | No non-participants were reported as present during interviews. |
|  | 16 | Description of sample: What are the important characteristics of the sample? | Adults with lived experience of primary craniospinal tumour pathways, including chordoma, chondrosarcoma, osteosarcoma and other craniospinal sarcoma pathways. Participants included people who had donated tissue, were unsure whether donation had been discussed, or reported a missed opportunity to donate. All interviewees were recruited from survey respondents who opted in to further contact. |
| Data collection | 17 | Interview guide: Were questions, prompts, guides provided by the authors? Was it pilot tested? | A semi-structured interview guide was used. It explored route to diagnosis/treatment, tissue-donation experience or missed opportunity, timing and framing of consent, information needs, trust, governance, family involvement and views on digital resources. The interview guide is described in the manuscript; it is not provided as a separate supplementary file for this submission. |
|  | 18 | Repeat interviews: Were repeat interviews carried out? | No repeat interviews were conducted. |
|  | 19 | Audio/visual recording: Did the research use audio or visual recording? | Interviews were audio-recorded with consent and professionally transcribed. |
|  | 20 | Field notes: Were field notes made during and/or after the interview or focus group? | Reflexive memos were used during analysis. Field notes during/immediately after interviews are not reported as a separate data source. |
|  | 21 | Duration: What was the duration of interviews or focus groups? | Interviews lasted approximately 45-60 minutes. |
|  | 22 | Data saturation: Was data saturation discussed? | Recruitment and sampling adequacy were discussed in relation to depth, specificity and information-richness. Eight interviews were completed and analysed to address the focused qualitative aim and inform subsequent co-design work; this is reported as analytic sufficiency rather than a statistical claim. |
|  | 23 | Transcripts returned: Were transcripts returned to participants for comment and/or correction? | Transcripts were not returned to participants for comment or correction. |
| **Domain 3: Analysis and findings** |  |  |  |
| Data analysis | 24 | Number of data coders: How many data coders coded the data? | Coding and theme development were led by the lead researcher and discussed with an experienced qualitative researcher. Reflexive thematic analysis does not require inter-coder reliability; theme development was supported by reflexive memoing and supervisory discussion. |
|  | 25 | Description of the coding tree: Did authors provide a description of the coding tree? | Yes. An Excel-based codebook and thematic development file is provided as Supplementary File 1, showing movement from initial codes to focused codes, subthemes, candidate themes and final analytic themes. |
|  | 26 | Derivation of themes: Were themes identified in advance or derived from the data? | Themes were generated inductively and interpretively from participant accounts through reflexive thematic analysis. |
|  | 27 | Software: What software, if applicable, was used to manage the data? | An Excel-based coding framework was used as a data-management aid to organise transcripts, codes, analytic notes and theme-development materials. Theme generation remained an interpretive process led by the researcher. |
|  | 28 | Participant checking: Did participants provide feedback on the findings? | Participants did not provide formal feedback on the findings in this qualitative phase. Their accounts informed subsequent consent-support and co-design work. |
| Reporting | 29 | Quotations presented: Were participant quotations presented to illustrate themes/findings? | Yes. Participant quotations are presented throughout the Results section and identified using study IDs PIC001-PIC008. |
|  | 30 | Data and findings consistent: Was there consistency between data presented and findings? | Yes. Findings are supported by direct quotations and by a supplementary codebook/thematic development file linking initial codes, subthemes, final analytic themes and the supportive layer reported as an overall observation. |
|  | 31 | Clarity of major themes: Were major themes clearly presented? | Yes. Four themes are clearly presented under the overarching analytic thread of making sense of tissue donation across the rare tumour pathway: “Why wouldn’t you?”; “Everything else becomes white noise”; “Once that’s been absorbed”; and “The thing that is most useful is you guys”. The Results section also reports a supportive layer across the pathway as an overall observation about support sought during participants’ experiences, comprising revisitable digital resources, paper options, clinician discussion and peer/family support. |
|  | 32 | Clarity of minor themes: Is there a description of diverse cases or minor themes? | Yes. The manuscript reports variation and divergent cases, including same-day consent being acceptable for one participant, uncertainty about whether donation was discussed, preference for paper options, digital support as an optional adjunct rather than a replacement for clinician discussion, and missed opportunity to donate. |
