# Supplementary material for: ‘A No‐Brainer, but Not a Simple Decision’: A Qualitative Study of Patient Experiences of Tissue Donation for Research in Rare Craniospinal Tumour Pathways
Source: Health Expect. 2026 Aug 22;29(4):e70839. doi: 10.1111/hex.70839 (PMC13499010; doi:10.1111/hex.70839)
Supplement: Supplementary file 3 [file HEX-29-e70839-s003.docx]

**Supplementary File 3. Semi-structured interview guide**

*This supplementary file reproduces the semi-structured interview guide used for the qualitative interview phase; formatting has been standardised for journal submission and the substantive question wording is unchanged.*

**Opening script, welcome + scene setting:**

1. Can you tell me about to your time to treatment?
2. As part of your treatment journey, you agreed to donate tissue for research?…

What was your motivation / reasoning to agree to donate?

3. Would there have been any factors that would have changed your decision not to donate?

**Time / Place / Resources:**

4. Where were you consented to donate tissue?
5. Did you feel the place was appropriate? If your opinion how could the process be improved?
6. Did you feel that you had sufficient time to read and understand the information provided? What could have been done differently...
7. If you were provided with information beforehand: would you (i) read it? (ii) Do you think this would be useful?

**Online / Digital:**

8. I am planning to build an online platform to provide patients with information about donating tissue for research, as well as info about participating in other research projects…

Do you think this would be useful? If so, what would you think we should include?

**Next steps + Thank you**

9. An important part of building this platform, is that it is useful and user friendly.

To ensure that future patients would want to use and engage with such a portal,

I would like to invite you to provide some feedback once the pilot platform is built.

Would this be something I could share with you later in time?
